# Soil Nitrogen Mineralization Is Driven by Functional Microbiomes Across a North–South Forest in China

**DOI:** 10.3390/microorganisms13122799

**Published:** 2025-12-09

**Authors:** Hongyan Cheng, Minshu Yuan, Chengjie Ren, Fazhu Zhao, Jun Wang

**Affiliations:** 1 Shaanxi Key Laboratory of Earth Surface System and Environmental Carrying Capacity, College of Urban and Environmental Science, Northwest University, Xi’an 710127, China; 2 Yangling Xinhua Ecology Technology Co., Ltd., Yangling 712100, China; 3 College of Agronomy, Northwest A&F University, Yangling 712100, China; 4 Shaanxi Key Laboratory for Carbon Neutral Technology, Northwest University, Xi’an 710127, China

**Keywords:** forest biomes, soil N mineralization rate, microbial N-cycling traits, denitrification pathway, nitrification pathway

## Abstract

Nitrogen (N) mineralization is a complex microbial-driven process that controls the supply of N for plants and microbes. The relative contribution of different microbial N-cycling species/genes to the variation in N mineralization rate (NMR) across contrasting forest biomes was unclear. Here, we investigate the linkages between soil metagenomes and N mineralization rates across 10 contrasting forest biomes (covering temperate, subtropical, and tropical forests) along a 3425 km north–south forest in China. We found that the NMR was higher in subtropical forests, and the variation in NMR can be explained by climate and soil environments, particularly for soil substrate NH_4_^+^. Similar to NMR, microbial N-cycling genes/species were also higher in subtropical forests, suggesting that the higher microbial N-cycling traits in warm regions may drive higher NMR. We also quantified the contribution of microbial N-cycling gene pathways to NMR across forest biomes and found that the microbial N-denitrification pathway (genes like *norZ*, *narG*, *nirK*, and *norB*) and nitrification pathway (genes like *nxr*) explained more variation in NMR than other pathways, such as N ammonification. Collectively, our work demonstrates the importance of microbial N-cycling traits to explain soil N mineralization rates across forest biomes and suggests that this information can be used to help improve the management of the N cycle in forests across biomes.

## 1. Introduction

Nitrogen (N) is one of the most important elements that often limits the productivity of terrestrial ecosystems worldwide. N mineralization [1,2], controlled by microbial communities, is the fundamental process that regulates the entrance of available N for plants and microbes. This process is, moreover, essential to regulate carbon sequestration and soil fertility [3,4]. According to the previous studies [5,6], the mineralization of soil organic N into inorganic N contributes 58% N for plant production, and soil N turnover was integrated into biogeochemical models to better predict the global biogeochemical cycles. Therefore, identifying the drivers of N mineralization rate (NMR) is crucial for developing models to better understand carbon–climate feedback [7,8].

N mineralization is the result of multiple co-occurring microbial processes through organic matter decomposition, exchange with the atmosphere (nitrification and denitrification), and loss by nitrate leaching [1,2]. N mineralization is, moreover, influenced by multiple environmental factors such as substrate availability [9], climate (e.g., temperature and precipitation) [10], vegetation [4], and edaphic properties (e.g., clay, soil organic carbon, total nitrogen, C:N ratio) from local [11,12] and regional [13,14] to global scales [6,15,16]. Studies have linked N cycling to microbial biomass [6] and the abundance of individual functional genes [2]. However, a holistic view of the multiple soil genes associate with soil N mineralization and reflecting the myriad of microbial gene, species, and enzyme activities controlling soil N mineralization across biomes is still lacking [2,17]. Particularly, microbial functional genes, rather than taxonomic patterns, can be potential keys to understanding organic matter decomposition [18]. However, the linkages between microbial N-cycling genes and potential mineralization rates have not been validated across large spatial scales with contrasting climates and environmental factors.

Microbial-driven N transformation represents a key process in the terrestrial N cycle; three key processes (i.e., ammonification, nitrification, and denitrification) are especially important in this context [1]. Among these processes, some members of microbial species (e.g., *Comammox nitrospira* ssp. and *N. inopinata*) can catalyze both steps of mineralization [2]. Based on metagenomic surveys, the distribution of nitrifying and denitrifying communities is still comparatively limited; several studies have reported their presence in terrestrial subsurface [2,19]. Thus, a joint assessment of the influences of the aforementioned microbial N-cycling communities on soil nitrogen mineralization as well as of their specific responses to large scales with different forest biomes is required. This information is critical to quantify the contribution of mineralization and denitrification genes in explaining potential net N mineralization rates.

To address these knowledge gaps, we conducted a topsoil (0–10 cm) survey across 10 contrasting forests (covering temperate, subtropical, and tropical forests) along a 3425 km north–south forest in China. A metagenomic approach was used to explore the microbial mechanisms underlying soil N mineralization in forests across biomes. The purpose of this study was to determine how shifts in microbial N-cycling traits (ammonification, nitrification, and denitrification genes) affect NMR in forests. Temperate ecosystems with relatively lower temperatures were suggested to be more limited by N than tropical forests with higher temperatures [20], since higher temperatures could accelerate the metabolic activity of microorganisms and change functional genes in N cycling [1]. Therefore, we hypothesized that soil NMR would be higher in tropical/subtropical forests and largely depend on microbial N-cycling genes and species. Moreover, microbial N-cycling pathways (ammonification, nitrification, and denitrification genes) differed in forests across biomes with different environmental factors [2,10]. We also predict that different microbial N-cycling pathways with varying functional genes may respond differently to soil N mineralization ratio in forests across biomes. Therefore, the objectives were (1) to reveal the differences in N mineralization ratio across forests; (2) to explore the regulatory effects of microbial nitrogen-cycling genes on the nitrogen mineralization ratio; (3) and to disentangle the environmental factors that drive the N mineralization ratio through microbial N-cycling traits across forest biomes.

## 2. Materials and Methods

### 2.1. Study Area and Field Sampling

The study area encompassed 10 forest ecosystems along a 3425 km north–south forest spanning temperate, subtropical, and tropical forests in China (Table S1). All the sampling sites were located in well-protected national nature reserves to minimize the effect of anthropogenic disturbance. This gradient provides an ideal natural laboratory to explore the ecological processes, since the climate factors, soil properties, and microbial properties vary greatly among different forests (Tables S1 and S2). Along this gradient, the mean annual temperature (MAT) and mean annual precipitation (MAP) ranged from 3.1 to 23.15 °C and from 486 to 2266 mm, respectively. Soil bulk density (BD), sand, silt, clay, and pH change ranged from 0.79 to 1.4 g/cm^3^, from 12.51 to 89.35%, from 4.8 to 68.14%, from 0.14 to 64.3%, and from 4.9 to 8.12%, respectively. SOC content and C:N ratio were higher in the temperate forest soil but lower in tropical/subtropical forest soils and changed ranging from 19.85 to 96.80 g/kg.

Soil samples were collected between July and August 2019. At each site, three sampling plots (50 × 50 m) were randomly established. After removing the surface litter, topsoil (0–10 cm) was collected from nine random locations within each plot. Subsequently, in order to reduce soil heterogeneity in each plot, the soil samples were then combined as a composite sample. In total, 30 soil samples (10 forest biomes × 3 plot replicates) were collected as biological replicates for subsequent analysis. All soil samples were sieved (2 mm diameter), removing fine roots and other plant debris. These sieved samples were divided into two subsamples. One subsample was maintained at −20 °C until the start of microbial measurements. Another remaining soil sample was air-dried and used to analyze chemical and physical properties.

### 2.2. Analysis of Soil Properties

Soil pH was determined in a 1:2.5 soil-to-deionized water mixture and then analyzed by using a pH electrode (FE28-Standard, Mettler, Greifensee, Switzerland). SOC content was determined with the K_2_Cr_2_O_7_ oxidation method. Soil total N was extracted by digesting the soil sample with sulfuric acid by adding copper sulfate and potassium sulfate, while total P was extracted by digesting the soil sample with sulfuric acid and perchloric acid. The extracted solutions were analyzed for the N and P concentrations with an Auto Analyzer (BRAN+LUEBBE-AA3, Norderstedt, Germany). Soil bulk density (BD) was measured from the gravimetric weight of the core before and after oven drying at 105 °C for 24h and considering the individual core volume. Soil texture (i.e., sand, silt, and clay) was measured using the hydrometer method [21].

### 2.3. Determination of Potential Net N Mineralization Rate (NMR)

Potential net N mineralization rate was determined as the change in inorganic N before and after lab incubation as described in [22]. Briefly, fresh soil samples were adjusted to 60% water-holding capacity (on a 10 g dry soil basis) and extracted with 50 mL KCl (2 mol L^−1^) to determine the initial dissolved inorganic N concentrations. Another subset of fresh samples was incubated in the dark at 25 °C for one week. The water content of the soil was maintained at 60% water-holding capacity by a regular supply of deionized water during incubation. The potential net mineralization rates were calculated as the changes in inorganic N ( ${\mathrm{NH}}_{4}^{+}$, ${\mathrm{NO}}_{3}^{-}$) before and after incubation.

For a time interval, $∆t=t_{i+1}-t_{i}$

$$R_{amo}=\left[c{\left({NH}_{4}^{+}\right)}_{i+1}-c{\left({NH}_{4}^{+}\right)}_{i}\right]/∆t$$

$$R_{nit}=\left[c{\left({NO}_{3}^{-}\right)}_{i+1}-c{\left({NH}_{3}^{-}\right)}_{i}\right]/∆t$$

$$NMR=R_{amo}+R_{nit}$$

where $t_{i}$ and $t_{i+1}$ are the initial and post-incubation time and $c{\left({NH}_{4}^{+}\right)}_{i}$ and $c{\left({NH}_{4}^{+}\right)}_{i+1}$ are the mean ammonium N concentration in the intial and incubated samples, respectively. Similarly, $c{\left({NH}_{3}^{-}\right)}_{i}$ and $c{\left({NO}_{3}^{-}\right)}_{i+1}$ are the mean nitrate N concentrations in the initial and incubated samples, respectively. $R_{amo}\;and\;R_{nit}$ represent the net ammonification and nitrification rates, respectively. The unit for N mineralization rate is mg·kg^−1^·day^−1^.

### 2.4. DNA Extraction and Sequencing

Soil DNA was extracted in triplicate from 0.5 g of fresh soil sample using the FastDNA spin kit for soil (MP Biomedicals, Cleveland, OH, USA), following the manufacturer’s instructions. The quality and integrity of the DNA extracts were assessed using a NanoDrop 2000 spectrophotometer (Thermo Fisher Scientific, Waltham, MA, USA). To obtain sufficient DNA for the shotgun metagenomic sequencing and to guarantee the representation of forest soil, the three DNA extracts from the same soil sample were pooled. Thus, one composite DNA sample per plot (resulting in 30 samples in total) was used for library construction and sequencing. The metagenome libraries were sequenced on an Illumina HiSeq 2000 (Illumina, Inc., San Diego, CA, USA) to generate 150 bp paired-end reads at greater sequencing depth. The reads aligned to the human genome were removed, and the lengths were trimmed with Sickle [23]. All DNA sequencing can be found on the National Center for Biotechnology Information (NCBI) website.

### 2.5. Metagenomics Analysis

To account for differences in sequencing depth across samples, we randomly subsampled an equal number of high-quality reads from each sample (the minimum number of reads obtained from any sample) prior to downstream bioinformatic analyses to ensure comparability. Raw sequencing reads were processed to obtain quality-filtered reads for further analysis. Sequencing adapters were removed from sequencing reads using Cutadapt (v1.2.1) [24]. Low quality reads were trimmed using a sliding-window algorithm in fastp [25]. Taxonomic classifications of metagenomic sequencing reads from each sample were performed using Kaiju [26] in greedy-5 mode against an nr-derived database, which included proteins from archaea, bacteria, viruses, fungi, and microbial eukaryotes. Megahit (v1.1.2) [27] was used to assemble for each sample using the meta-large presented parameters [28]. The lowest common ancestor taxonomy of the non-redundant contigs was obtained by aligning them against the NCBI nucleotide database by BLASTN (https://blast.ncbi.nlm.nih.gov/Blast.cgi), and contigs assigned to Viridiplantae or Metazoa were removed from the following analysis. The annotation of contigs (longer than 200 bp) was performed using both MetaGeneMark [29] and MetaEuk [30] to predict genes. In particular, MetaEuk considered both prokaryotic and eukaryotic exons. According to the results of the KEGG database, the functional annotation and taxonomic assignment from each sample were obtained for further analysis. Based on the published literature [2], potential N-transforming microbial functional species/genes data are shown in Tables S3 and S4.

### 2.6. Statistical Analyses

Before the analysis, all the data were tested for normal distribution. We performed logarithmic transformation on non-normally distributed data. One-way analysis of variance (ANOVA) was performed to assess the effect of differences in forest biomes on the climatic factors (e.g., MAT and MAP), soil properties (pH, BD, sand, silt, clay, and SOC), soil substrates (TN, C:N ratio, NH_4_^+^, NO_3_^−^), and N-cycling functional species and genes with a 0.05 significance level. A network analysis was performed to explore the N-cycling species/genes. Redundancy analysis (RDA) was performed to identify the relationship between environmental variability (i.e., climate, soil properties, and substrates) and N-cycling species/genes. Random forest analysis was used to evaluate the importance of each functional gene for NMR with the ‘randomforest’ package. Regression analysis was used to reveal the relationship microbial functional genes group, potential N-transforming microbial functional species, and NMR.

The partial least squares path model (PLS-PM) was constructed to identify the direct and indirect drivers of NMR. The selection of variables was based on their established roles in controlling microbial activity and N cycling [13,16]. Specifically, MAT and MAP were selected as composite variables representing climatic factors. Soil pH, texture (sand, silt, clay content), and bulk density (BD) were chosen as indicators of soil properties that influence microbial habitat and physicochemical conditions. Soil C:N ratio, NH_4_^+^, and NO_3_^−^ concentrations were used as proxies for soil substrate availability and quality, which directly fuel microbial metabolism. The conceptual model illustrating the hypothesized causal relationships among these blocks of variables is provided in Figure S1. We assumed that the variation in climate will change the soil environment, soil substrate, and functional genes, and both of them will affect each other and have a different effect on soil NMR. In the PLS-PM analysis, we compared the model-implied variance–co-variance matrix against the observed variance–covariance matrix, and the data were fitted to the models using the maximum likelihood estimation method. All the analysis was conducted using the R statistical software v.4.0.2.

## 3. Results

### 3.1. Changes in Soil Substrates and Net N Mineralization Rates

**Figure 1:**
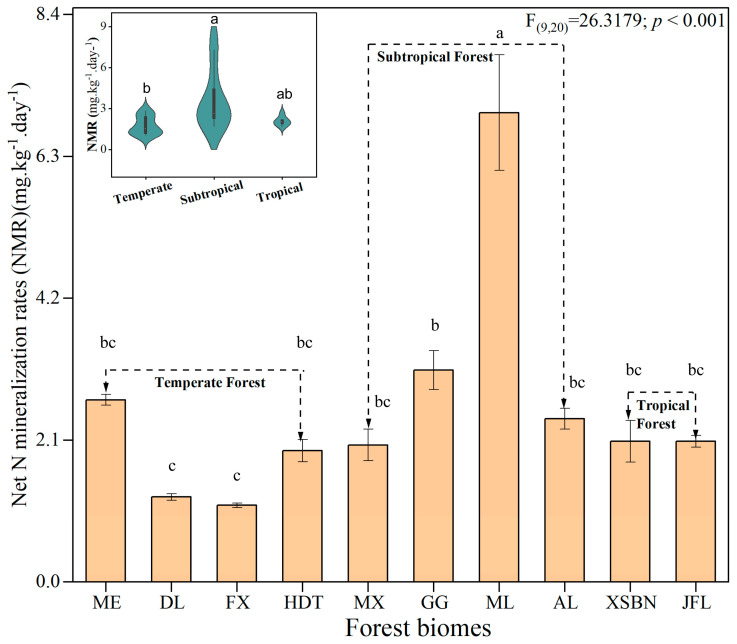
**Changes in soil N mineralization rate (NMR) across forest biomes.** Different letters indicate the significant level (*p* < 0.05). Maoer Mountain (ME), Dongling Mountain (DL), Fuxian (FX), Huoditang (HDT), Maoxian (MX), Gongga Mountain (GG), Mulun (ML), Ailao Mountain (AL), Xishuangbanna (XSBN), Jianfengling (JFL).

Except for the sample sites of ME, the soil TN, NH_4_^+^, and NO_3_^−^ were higher in subtropical forest soils and lower in temperate forest soils. Moreover, soil NMR ranged from 1.07 to 8.23 mg·kg^−1^·day^−1^ and was higher in subtropical forest soils but was lower in temperate forest soils (Figure 1). 

### 3.2. Changes in Microbial N-Cycling Genes and Species

**Figure 2:**
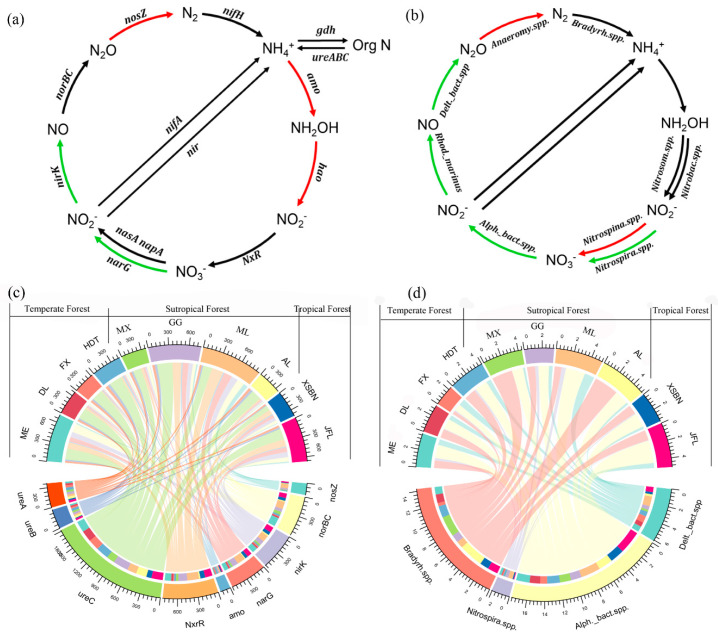
Conceptual diagrams show the key microbial functional species/genes in the nitrogen cycle across biomes. (**a**) Microbial nitrogen-transforming gene level, (**b**) potential nitrogen-transforming microbial networks (species level), (**c**) changes in different N-cycling genes, and (**d**) changes in different N-cycling genes in forests across biomes.

Soil microbial N (ammonification, nitrification, and denitrification) species/genes significantly changed across forest biomes and were higher in the subtropical forest than those in the temperate forest (Figure 2, Tables S2 and S3) (*p* < 0.05). Specifically, *Alphaproteobacteria bacterium* spp. and *Nitrospira* spp. accounted for a larger percentage and were higher in tropical/subtropical forest soils but lower in temperate forest soils. However, *Deltaproteobacteria bacterium* spp. and *Nitrosomonas* spp. showed a different trend, which was that they were higher in temperate forest soils but lower in tropical forest soils. Moreover, for the microbial N-cycling genes, except for sample sites at ME, the abundance of both denitrification and nitrification genes significantly decreased from the lower latitude with tropical/subtropical forest soils to the higher latitude with temperate forest soils (*p* < 0.05). Particularly, *norB*, *nirK*, *narG*, and *Nxr* accounted for a large percentage, changing from 3.74 to 166.84, 9.65 to 118.75, 4.92 to 155.81, and 8.65 to 224.33, respectively.

### 3.3. Microbial N-Cycling Traits Explain a Unique Portion of Variation in Soil NMR

**Figure 3:**
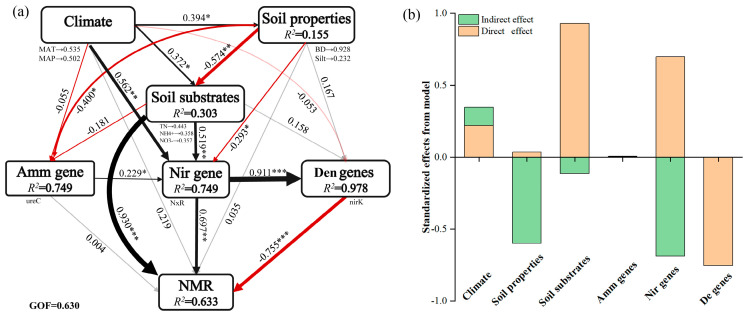
Effect of environmental viabilities on the N mineralization rate (NMR). (**a**,**b**) Directed graph of the partial least squares path model (PLS-PM) showing the effects of the climate, soil properties, soil substrates, and microbial N-cycling pathways (e.g., ammonification (amm), nitrification (nir), and denitrification (den)). Single-headed arrows indicate the hypothesized direction of causation. Indicated values are the path coefficients (*** when *p* < 0.001, ** when *p* < 0.05, and * when *p* < 0.01). Green arrows indicate a positive effect, whereas red arrows indicate a negative effect. Black and red arrows indicate positive and negative relationships, respectively. The arrow width is proportional to the strength of the relationship. Models with different structures were assessed using the goodness of fit statistic, a measure of the overall prediction performance. An a priori model associated with this figure is available in Figure S1.

**Figure 4:**
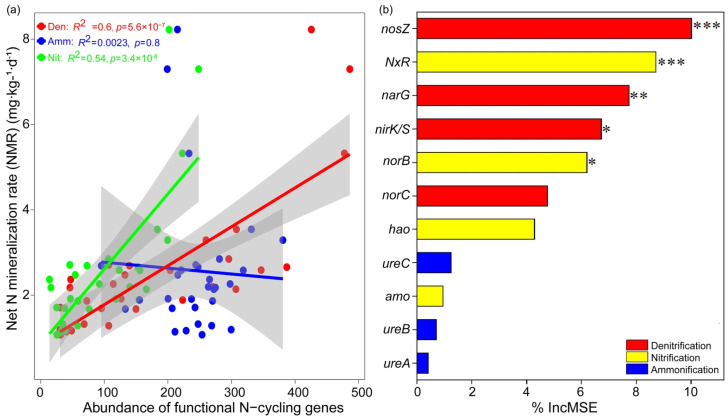
Contribution of different microbial N-cycling functional genes to N mineralization rate (NMR). (**a**) Regression analysis shows the different microbial N-cycling pathways (e.g., ammonification, nitrification, and denitrification) to NMR; (**b**) random forest analysis shows the significant microbial N-cycling functional genes for N mineralization rate (*** when *p* < 0.001, ** when *p* < 0.05, and * when *p* < 0.01).

**Figure 5:**
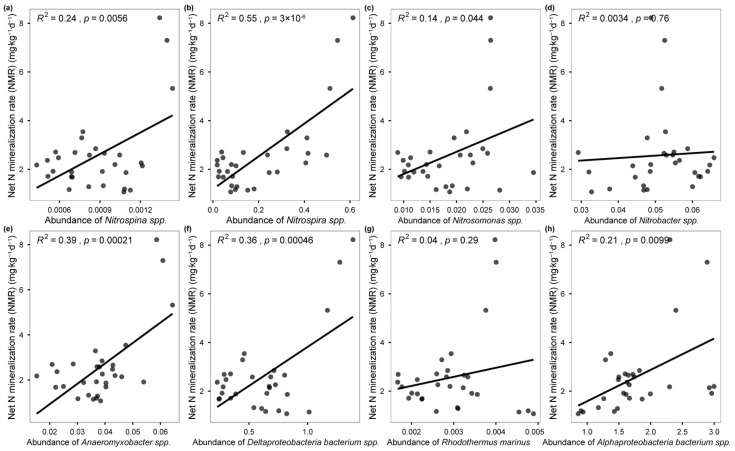
**Regression analysis shows the different microbial N-cycling species to NMR**. (**a**) *Nitrospina* spp. (**b**) *Nitrospira* spp. (**c**) *Nitrosomonas* spp. (**d**) *Nitrobacter* spp. (**e**) *Anaeromyxobacter* spp. (**f**) *Deltaproteobacteria bacterium* spp. (**g**) *Rhodothermus marinus.* (**h**) *Alpharoteobacteria bacterium* spp.

The PLS–PM analysis revealed that climate and soil environment (including soil properties and soil substrates) were the primary factors in regulating soil NMR, and the microbial N-cycling genes/species directly determined soil NMR through changing soil substrates (e.g., NH_4_^+^ and NO_3_^−^) and climate (e.g., MAT and MAP) (Figure 3a). The analysis of direct effect size and regression analysis further showed that microbial denitrification and nitrification, particularly for the denitrification pathway, rather than ammonification pathways explain more N mineralization rates across forest biomes (Figure 3b and Figure 4a). The random forest analysis also showed that four denitrification genes (*norZ*, *narG*, *nirK*, *norB*) and one nitrification gene (*nxr*) were the most important for soil NTR across forest biomes (Figure 4b). Correspondingly, the NMR was significantly correlated with the abundance of *Nitrospina* spp. (*R*^2^ = 0.24, *p* = 0.0056), *Nitrospira* spp. (*R*^2^ = 0.55, *p* = 3 × 10^−6^), *Nitrosomonas* spp. (*R*^2^ = 0.14, *p* = 0.044), *Anaeromyxobacter* spp. (*R*^2^ = 0.39, *p* = 0.00021), *Deltaproteobacteria bacterium* spp. (*R*^2^ = 0.36, *p* = 0.00046), and *Alphaproteobacteria bacterium* spp. (*R*^2^ = 0.21, *p* = 0.0099) (Figure 5).

## 4. Discussion

### 4.1. Soil N Mineralization Ratio in Forests Across Biomes

Our results showed that NMR was higher in the subtropical/tropical forests than that in the temperate forest (Figure 1). Similar with our result, Li Z, Zeng Z, Tian D, Wang J, Wang B, Chen HYH, Quan Q, Chen W, Yang J, Meng C, Wang Y, and Niu S [6] revealed a clear latitudinal pattern of soil NMR, which was high in low latitudes but low in high latitudes. The patterns of NMR may be attributed to the indirect effects of climate and vegetation and direct influence of soil substrates and soil microorganisms [6,12,31] (Figure 4). For example, it has been reported that MAT legacies are critical for informing soil NMR at local or regional scales [15,31]—likely because of the link with organic matter content. Previous studies pointed out that the crucial factor determining NMR is its stimulation of microbial N cycling [2,20], therefore, potentially affecting the activities of the proteolytic enzyme in N cycling [1,32]. We also describe in our study that climate was the primary driver of NMR (Figure 3). Furthermore, at the regional scale, the important role of soil substrate availability (e.g., NH_4_^+^ and NO_3_^−^) for the regulation of N ammonification is well accepted [11,16] and confirmed by the findings of the present study (Figure 3). This is because the soil substrate can induce a change in microbial N-cycling traits at the regional scale [1,31,33], ultimately resulting in changes in soil NMR (Figure 3).

### 4.2. Spatial Patterns of Microbial N-Cycling Traits and Their Controls on Soil NMR in Forests Across Biomes

Similar to what we found with NMR, we found that the proportion of microbial N-cycling genes/species was higher in the subtropical forest than that in the temperate forest (Figure 2) and showed a positive correlation with NMR (Figure 3). This response is consistent with a previous study reporting that the highest frequencies of microbial N-cycling pathways were detected in tropical/subtropical forests, whereas the lowest frequency was observed in cold regions [1]. Our findings further indicated that soil substrate, such as NH_4_^+^, changed along climate gradients and was the primary driver of soil microbial N-cycling traits in forests across biomes (Figure 3 and Figure S2), confirming the previous results of Lal R [34]. However, in contrast to our finding, a previous study indicated a high tolerance of the nitrification process to temperature in a managed grassland [17]. Especially, a meta-analysis observed no significant effects of elevated temperature on grassland microbial N-cycling processes [15,35]. This discrepancy could be explained by the differances in vegetation types and soil environment under different ecosystems. Specifically, diverse plant communities and their subsequent changed plant productivity can increase soil microbial biomass and activities because of a greater amount of plant-derived food and expanded microbial niches [36] and are more likely to enhance mineralization rates of soil N [37,38]. Thus, forest biomes in tropical/subtropical ecosystems with higher net primary productivity were observed [39] and may also contribute to soil microbial N cycling through plant-derived components. It has also been reported that soils with higher moisture could favor N denitrification and associated N loss in gaseous forms (for example, N_2_, N_2_O) in wetter sites [2], thus confirming the higher microbially driven NMR in tropical forest (higher MAP, Table S1) soils than that in temperate forest soils.

We further provided evidence of the relative contribution of different N gene pathways to explain NMR and found that NMR across forest biomes was largely driven by microbial nitrification/denitrification pathways rather than ammonification pathways. Particularly, the microbial denitrification pathway was especially important to explain NMR (Figure 4). These findings correspond to a widely accepted viewpoint that denitrification is one of the biochemical pathways of nitrous oxide (N_2_O) production and may in fact be responsible for the greater proportion of total nitrifier-induced N_2_O production [2,33,40]. Yoon S, Cruz-García C, Sanford R, Ritalahti KM, and Löffler FE [41] further explained that denitrification dominated at a low soil C:N ratio, whereas ammonification was the predominant product at a high C:N ratio in soil, and incubation above pH 7.0 may favor ammonium formation. Thus, the lower C:N in tropical/subtropical forests and a pH lower than 7.0 (Table S2) may stimulate denitrification pathways for changed soil NMR. However, our finding is contrary to a previous study [17], showing that nitrification is a fundamental process in terrestrial N cycling, and a shift in nitrifier community structure could potentially result in higher N_2_O emission rates at the end of the drought region. These differences could be induced by the following two aspects. On the one hand, the denitrification process, in which bacteria reduce N in NO_3_^−^ to N_2_, results in the loss of soil N, while nitrification is the conversion of ammonium to nitrate, which is the turnover of the internal elements of N instead of direct N loss [1,2]. On the other hand, the proven viewpoint showed that the denitrification process is an anaerobic condition and favors warm and wet regions [42,43], while the nitrification process occurs under oxygen conditions and likes a ventilated environment [17,44]. Under such circumstances, larger variations in climate conditions (e.g., MAT and MAP) across wide forest biomes (Table S1) may result in differential responses of microbial N mineralization genes/species to soil NMR. Overall, our study delivers novel findings highlighting the importance of microbial functional traits for driving the soil NMR across wide forest biomes.

We further seek to link particular N-cycle driven species with N mineralization rates. Several abundant species and genes were observed to change across the forest biomes and showed different responses with soil NMR (Figure 2 and Figure 3). Specifically, the presence of numerous established microbial denitrification genes, such as *norZ*, *narG*, *nirK*, and *norB*, also changed with soil NMR, confirming their role in the soil N cycling [2,45]. Yoon S, Cruz-García C, Sanford R, Ritalahti KM, and Löffler FE [41] reported that lower C:N ratios in soil increased transcription levels of denitrification genes (*nirK* and *nosZ*), leading to the predominance of denitrification; thus, the tropical/subtropical forest soils with a lower C:N ratio may contribute a higher abundance of these denitrification genes, thereby stimulating higher NMR. In addition, the discovered *Nitrospira* spp. and their corresponding nxr gene can oxidize ammonia all the way to nitrate [44]; thus, the higher abundance of *Nitrospira* spp. in subtropical forest soils drives higher NMR (Figure 2 and Figure 5). Denitrification species such as *Deltaproteobacteria bacterium* spp. also accounted for large variation in NMR across forest biomes, since this species harbors the critical step that oxidize to the production of nitrous oxide (N_2_O), which was considered as the end-products of denitrification [2]. Collectively, these results provide evidence for the importance of microbial function for soil N loss [2,17,46] and predict that the subtropical forest with relatively higher N-nitrification/denitrification species may stimulate N mineralization rate in soil through increasing their corresponding microbial nitrification/denitrification genes, further regulating soil N dynamics in these regions.

## 5. Conclusions

Despite that the climate and soil substrates were the primary drivers of the patterns of soil NMR—higher in subtropical/tropical forests than that in temperate forests—we also provided solid evidence of significant correlations between the proportion of N genes and N and further suggested that higher microbial N denitrification and nitrification genes in subtropical forest soils may drive the higher ratio of NMR across biomes. Notably, soil microbial N-denitrification genes differed greatly across forest biomes and, together with nitrification, drove N mineralization rates. Ammonification genes were not significantly correlated with this process, suggesting that this step might not be a limiting pathway in the mineralization of N in soil. Collectively, our findings highlight the importance of microbial functional traits for soil potential net N mineralization rates and advance our mechanistic understanding of the global soil N cycle across contrasting forest biomes. However, the relatively small sample size from the small watershed may have influenced the results. Larger-scale spatial sampling in future studies could further strengthen our mechanistic understanding of the soil N cycle.

## Data Availability

The original contributions presented in this study are included in the article/Supplementary Materials. Further inquiries can be directed to the corresponding author.

## Supplementary Materials

The following supporting information can be downloaded at: https://www.mdpi.com/article/10.3390/microorganisms13122799/s1, Table S1: Background information for the 10 sampling sites across forest biomes; Table S2: Changes in soil characteristics across forest biomes; Table S3: Abundance of microbial N-cycling species (average values and standard error) across the forest biomes. Table S4: Abundance of microbial N-cycling genes (average values and standard error) across the forest biomes. Figure S1: A priori model showing the rationale behind the direct and indirect effects of climate (MAT and MAP), soil properties (pH, silt, clay, sand, and BD), soil substrates (TN, C:N, NH_4_^+^, and NO_3_^−^), and microbial N-denitrification and nitrification species/genes on the soil NMR. Figure S2: Redundancy analysis (RDA) was performed to identify the relationship between environmental variabilities (i.e., climate, soil properties, and substrates) and N-denitrification and nitrification species (a)/genes (b). Figure S3: Shift in soil cumulative priming effect across forest biomes. Maoer Mountain (ME), Dongling Mountain (DL), Fuxian (FX), Huoditang (HDT), Maoxian (MX), Gongga Mountain (GG), Ailao Mountain (AL), Xishuangbanna (XSBN), Mulun (ML), Jianfengling (JFL).

## Abbreviations

The following abbreviations are used in this manuscript:

NMR	N mineralization rates
SOC	soil organic C
TN	total N
MAT	mean annual temperature
MAP	mean annual precipitation
BD	soil bulk density
