# Supplementary material for: Soil Nitrogen Mineralization Is Driven by Functional Microbiomes Across a North–South Forest in China
Source: Microorganisms. 2025 Dec 9;13(12):2799. doi: 10.3390/microorganisms13122799 (PMC12736190; doi:10.3390/microorganisms13122799)

Supplementary information

Soil nitrogen mineralization is driven by functional microbiomes across a north–south forest transects in China

Hongyan Cheng [1,2], Minshu Yuan [1], Chengjie Ren [3], Fazhu Zhao[1,4] and Jun Wang[1,4,*]

1 Shaanxi Key Laboratory of Earth Surface System and Environmental Carrying Capacity, College of Urban and Environmental Science, Northwest University, Xi'an 710127, China
2 YanglingXinhua Ecology Technology co.,ltd, Yangling, 712100 Shaanxi, China
3 College of Agronomy, Northwest A&F University, Yangling, 712100 Shaanxi, China
4 Shaanxi Key Laboratory for Carbon Neutral Technology, Northwest University, Xi'an 710127, China
* Correspondence: wangj@nwu.edu.cn

Table S1 Background information for the 10 sampling sites across forest biomes

Sites No.	Forest site	Latitude (°)	Longtitude (°)	MAP (mm)	MAT (℃)	Climate types
1	ME	45.41	127.71	629	3.10	Temperate continental climate
2	DL	39.97	115.43	650	5.00	Warm temperate semi-humid climate
3	FX	36.11	109.63	580	7.40	Warm temperate climate
4	HDT	33.43	108.45	1023	9.00	North subtropical humid climate
5	MX	31.79	104.71	486	11.00	North subtropical humid climate
6	GG	29.65	102.11	1000	13.00	Subtropical climate
7	ML	25.13	108.00	1529	19.38	Subtropical climate
8	AL	23.87	103.51	1086	18.30	Subtropical humid climate
9	XSBN	21.83	101.20	1500	21.60	Tropical climate
10	JFL	18.71	108.91	2266	23.15	Tropical rainforest climate

Maoer Mountain (ME), Dongling Mountain (DL), Fuxian (FX), Huoditang (HDT), Maoxian (MX), Gongga Mountain (GG), Ailao Mountain (AL), Mulun (ML), Xishuangbanna (XSBN), Jianfengling (JFL). MAT, mean annual temperature; MAP, mean annual precipitation

Table S2. Changes of soil characstersitcs across forest biomes. Different letters indicated the significant level ($p<0.05$). Maoer Mountain (ME), Dongling Mountain (DL), Fuxian (FX), Huoditang (HDT), Maoxian (MX), Gongga Mountain (GG), Mulun (ML), Ailao Mountain (AL), Xishuangbanna (XSBN), Jianfengling (JFL).

	ME	DL	FX	HDT	MX	GG	ML	AL	XSBN	JFL	$F_{(9, 20)}$	*P*
**pH**	5.02±0.04f	6.85±0.01b	8.12±0a	5.9±0de	5.6±0.13e	6.2±0cd	6.71±0.35b	5.94±0.05cde	5.07±0.09f	6.3±0.01c	56.685	<0.001***
**BD(g cm^{-3})**	0.94±0ef	1.02±0.06de	0.9±0fg	1.2±0b	1.1±0cd	0.81±0g	0.83±0.04g	1.12±0.04bc	1.08±0cd	1.32±0.05a	28.729	<0.001***
**Sand(%)**	63.77±3.18b	89.35±0a	14.79±1.9f	49.6±0c	26.33±0.54e	33.07±1.44d	14.45±0.33f	24.02±0.88e	47.7±0.13c	50.98±0c	328.992	<0.001***
**Silt(%)**	23.93±2.09e	9.65±0f	60.93±1.81a	49.87±0b	64.14±2.13a	4.97±0.12g	64.56±0.34a	62.58±0.83a	29.53±0.32d	41.22±0c	401.696	<0.001***
**Clay(%)**	12.3±1.18de	1±0g	24.28±1.08b	0.53±0g	9.52±1.99ef	61.97±1.33a	20.99±0.21c	13.4±1.48d	22.78±0.29bc	7.8±0f	295.381	<0.001***
**SOC(g kg^{-1})**	99.33±6.04a	46.21±5.54bc	39.37±5.74bcd	40.95±4.55bcd	28.6±2.83de	38.92±6.08bcd	52.89±5.97b	36.37±2.87cd	20.37±0.37e	33.75±0.6cde	21.981	<0.001***
**TN(g kg^{-1})**	3.17±0.14b	1.4±0.13e	1.52±0.17e	1.9±0.11de	1.61±0.07e	2.28±0.1cd	4.72±0.48a	2.56±0.2c	1.74±0.12de	2.31±0.1cd	25.728	<0.001***
**C:N**	31.34±0.56ab	33.71±6.03a	27.45±7.29abc	21.53±2bcd	17.67±1.17cd	17.06±2.61d	11.2±0.51d	14.23±0.01d	11.83±0.87d	14.67±0.79d	6.373	<0.001***
**NH_4^+(mg kg^{-1})**	33.34±0.66ab	17.05±2.73d	25.36±2.06c	27.44±5.09bc	27.66±0.66bc	28.28±4.31bc	40.16±1.15a	20.68±0.52cd	24.89±0.37c	23.61±0.13cd	7.048	<0.001***
**NO_3^-(mg kg^{-1})**	22.64±2.38a	14.19±0.99b	6.87±2.1c	6.27±3.37c	11.97±0.59b	13.26±1.13b	26.45±0.57a	14.11±1.9b	14.03±0.97b	6.68±0.23c	15.236	<0.001***

Table S3 **Abundance of microbial N cycling species (average values and standard error) across the forest biomes.** Different letters indicate significant differences (ANOVA, P < 0.05, Tukey’s HSD post-hoc analysis) among different land use types. *, P<0.05; **, P<0.01.

	phyla level	Species level	ME	DL	FX	HDT	MX	GG	ML	AL	XSBN	JFL	*F*（9，20）	*P*
Denitrification	Proteobacteria	Anaeromyxobacter spp	0.0396±0.0017bc	0.0338±0.00204c	0.037±0.00076c	0.0348±0.00491c	0.04±0.00168bc	0.0406±0.00343bc	0.0613±0.00166a	0.0221±0.00381d	0.0251±0.0036d	0.0479±0.00312b	14.123	<0.001***
	Proteobacteria	Deltaproteobacteria bacterium spp	0.7345±0.03576bc	0.5969±0.02955cd	0.8627±0.07739b	0.6319±0.14717cd	0.2734±0.01648f	0.4779±0.02566de	1.2771±0.06286a	0.2836±0.03283f	0.3064±0.04219ef	0.6666±0.00726cd	25.348	<0.001***
	Bacteroidetes	Rhodothermus marinus	0.003±0.00013c	0.0029±0.00017c	0.0047±0.0001a	0.0027±0.00047cd	0.0021±0.00007de	0.003±0.00018c	0.0039±0.00008b	0.002±0.00029e	0.002±0.00017e	0.0032±0.00015c	16.004	<0.001***
	Proteobacteria	Alphaproteobacteria bacterium.spp	1.7374±0.05104cd	1.3609±0.0931e	0.8998±0.02204f	1.648±0.01752cde	1.4427±0.09311de	1.387±0.06129e	2.5297±0.18178b	1.8409±0.22838c	1.8607±0.08006c	2.9607±0.02126a	30.529	<0.001***
Nitrification	Nitrospinae	Nitrospina spp	0.0009±0.00005bcd	0.0008±0.00007cde	0.0011±0.00002b	0.0009±0.00021bcd	0.0006±0.00004ef	0.0008±0.00002cde	0.0014±0.00003a	0.0005±0.00004f	0.0007±0.00001def	0.001±0.00009bc	10.149	<0.001***
	Nitrospirae	Nitrospira spp	0.3262±0.05117bc	0.0947±0.00683e	0.1366±0.03058de	0.2461±0.09251cd	0.0317±0.00613e	0.4114±0.04964b	0.5569±0.02967a	0.025±0.00644e	0.1532±0.06897de	0.0817±0.01082e	15.206	<0.001***
	Proteobacteria	Nitrosomonas spp	0.0245±0.00096ab	0.0178±0.00088bcd	0.023±0.00276ab	0.0241±0.00578ab	0.0103±0.00052e	0.0207±0.00067abc	0.0264±0.00002a	0.0119±0.00156de	0.0106±0.0009e	0.0149±0.00093cde	7.922	<0.001***
	Proteobacteria	Nitrobacter spp	0.0552±0.00186abc	0.0515±0.00425bc	0.0391±0.00413de	0.0592±0.00194ab	0.064±0.00105a	0.0512±0.00168bc	0.051±0.00113bc	0.0587±0.003ab	0.0353±0.00472e	0.0471±0.00159cd	9.828	<0.001***

Table S4 Abundance of microbial N cycling genes (average values and standard error) across the forest biomes. Different letters indicate significant differences (ANOVA, P < 0.05, Tukey's HSD post-hoc analysis) among different land use types. *, P<0.05; **, P<0.01.

pathway	Gene name	ME	DL	FX	HDT	MX	GG	ML	AL	XSBN	JFL	*F*（9，20）	*P*
Ammonification	***ureC***	192.49±12.15b	191.34±16.98b	189.5±16.67b	186.97±18.85b	140.87±9.11b	263.33±13.86a	156.69±6.75b	180.21±2.44b	89.2±12.77b	192.03±6.19b	12.383	<0.001**
	ureB	26.28±2.02abc	25.24±2.12abc	26.7±4abc	39.13±2.02a	31.62±2.18ab	37.61±1.67a	23.35±0.47bc	37.7±5.96a	14.43±1.33c	29.81±3.09ab	7.084	<0.001**
	ureA	31.08±4.66a	31.75±4.57a	38.58±6.33a	44.16±0.91a	34.98±2.76a	42.59±3.64a	35.7±4.54a	43.44±4.01a	24.28±3.62a	35.78±2.32a	2.45	0.046*
Denitrification	***nosZ***	19.23±2.67bcd	2.24±1.22e	1.75±0.16e	3.34±1.66e	6.48±1.73e	24.85±5.62b	75.58±2.15a	8.72±4.32de	10.97±6.25cde	20.88±2.66bc	42.038	<0.001***
	norB	144.44±14.85a	24.13±4.92ef	5.71±1.15f	28.44±7.61def	51.15±8.37cd	51.86±8.44cd	117.16±2.39b	15.5±2.55f	46.71±10.67cde	69.74±5.21c	33.072	<0.001***
	norC	8.5±1.04ab	1.22±0.27de	1.88±0.26cde	2.28±1.05cde	0.45±0.38e	4.33±1.08cd	10.74±1.64a	1.54±1.27de	5.42±1.22bc	7.94±1.86ab	9.946	<0.001***
	nirS	0.08±0.08b	0±0b	0.12±0.12b	0.39±0.23b	0.84±0.42b	2.24±1.16b	7.6±2.26a	0.49±0.44b	0.04±0.04b	0.81±0.29b	7.739	<0.001***
	nirK	95.04±3.33ab	23.94±9.5d	14.31±1.8d	19.69±5.8d	30.56±3.04d	74.75±7.86bc	113.79±2.55a	24.91±6.33d	56.1±14.5c	92.5±8.4ab	25.308	<0.001***
	narG	75.8±8.84bc	23.12±4.73e	10.29±2.09e	18.28±7.72e	28.72±3.61de	99.1±8.1b	138.15±11.76a	15.62±6.65e	51.64±14.04cd	79.6±7.34b	27.089	<0.001***
Nitrification	***hao***	2.01±0.4bc	0.38±0.38d	0.41±0.21d	0.83±0.36cd	0±0d	5.65±0.8a	2.59±0.65b	0±0d	0.48±0.48d	0±0d	18.076	<0.001***
	amo	15.85±1.64bc	7.79±1.28cde	14.86±1.54bcd	28.11±6.87a	4.64±0.24de	22.32±4.16ab	22.57±2.22ab	1.08±0.83e	18.46±4.2abc	16.69±4.45bc	6.325	<0.001***
	NxR	109.67±11.74cd	35.56±5.78e	15.16±3.05e	29.69±12.15e	41.54±4.67e	151.66±13.65b	199.17±15.19a	24.15±9.45e	82.26±22.11d	124.91±9.86bc	27.372	<0.001***

Figure S1. A priori model showing the rationale behind the direct and indirect effects of Climate (MAT and MAP), soil properties (pH, silt, clay, sand, and BD), soil substrates (TN, C: N, NH_4^+, and NO_3^-), and microbial N- denitrification and nitrification species/genes on the soil NMR.

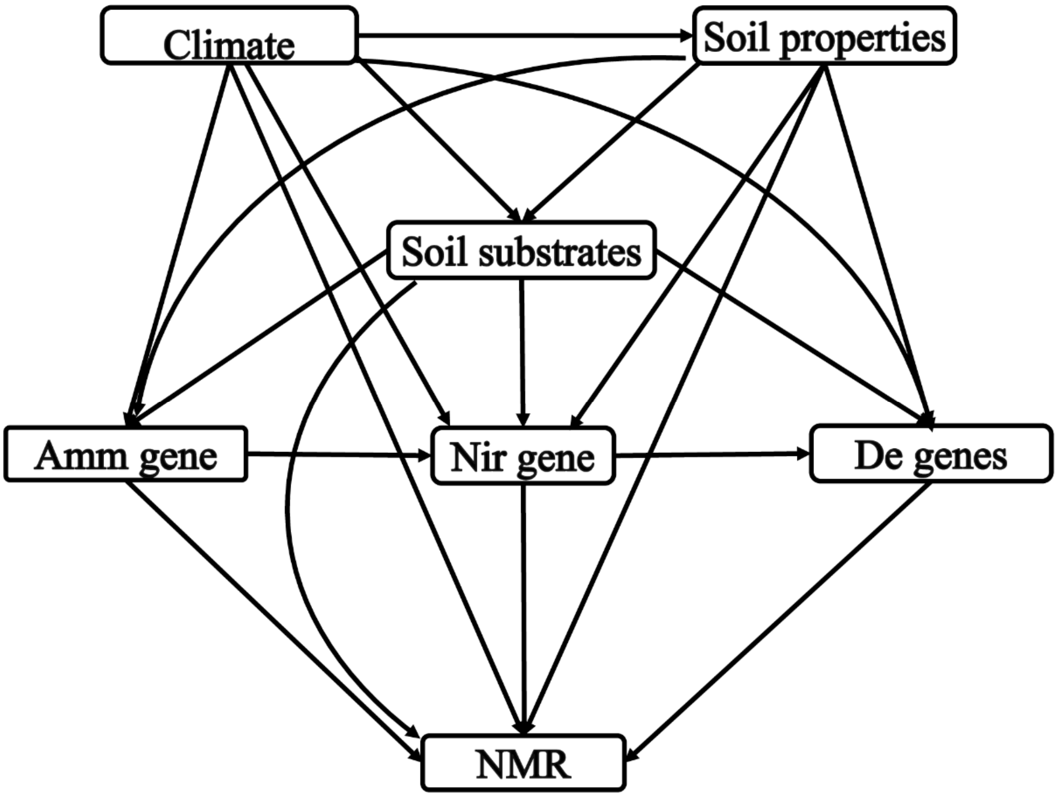

Figure S2 Redundancy analysis (RDA) was performed to identify the relationship between environmental variabilities (i.e., climate, soil properties, and substrates) and N- denitrification and nitrification species (a) /genes (b).

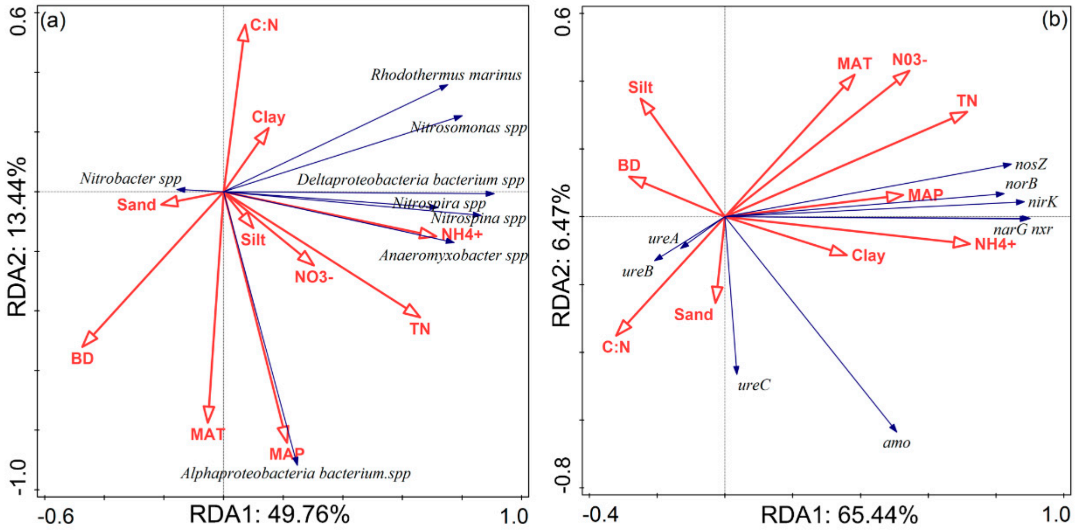

Figure S3 Shift in soil cumulative priming effect across forest biomes. Maoer Mountain (ME), Dongling Mountain (DL), Fuxian (FX), Huoditang (HDT), Maoxian (MX), Gongga Mountain (GG), Ailao Mountain (AL), Xishuangbanna (XSBN), Mulun (ML), Jianfengling (JFL).

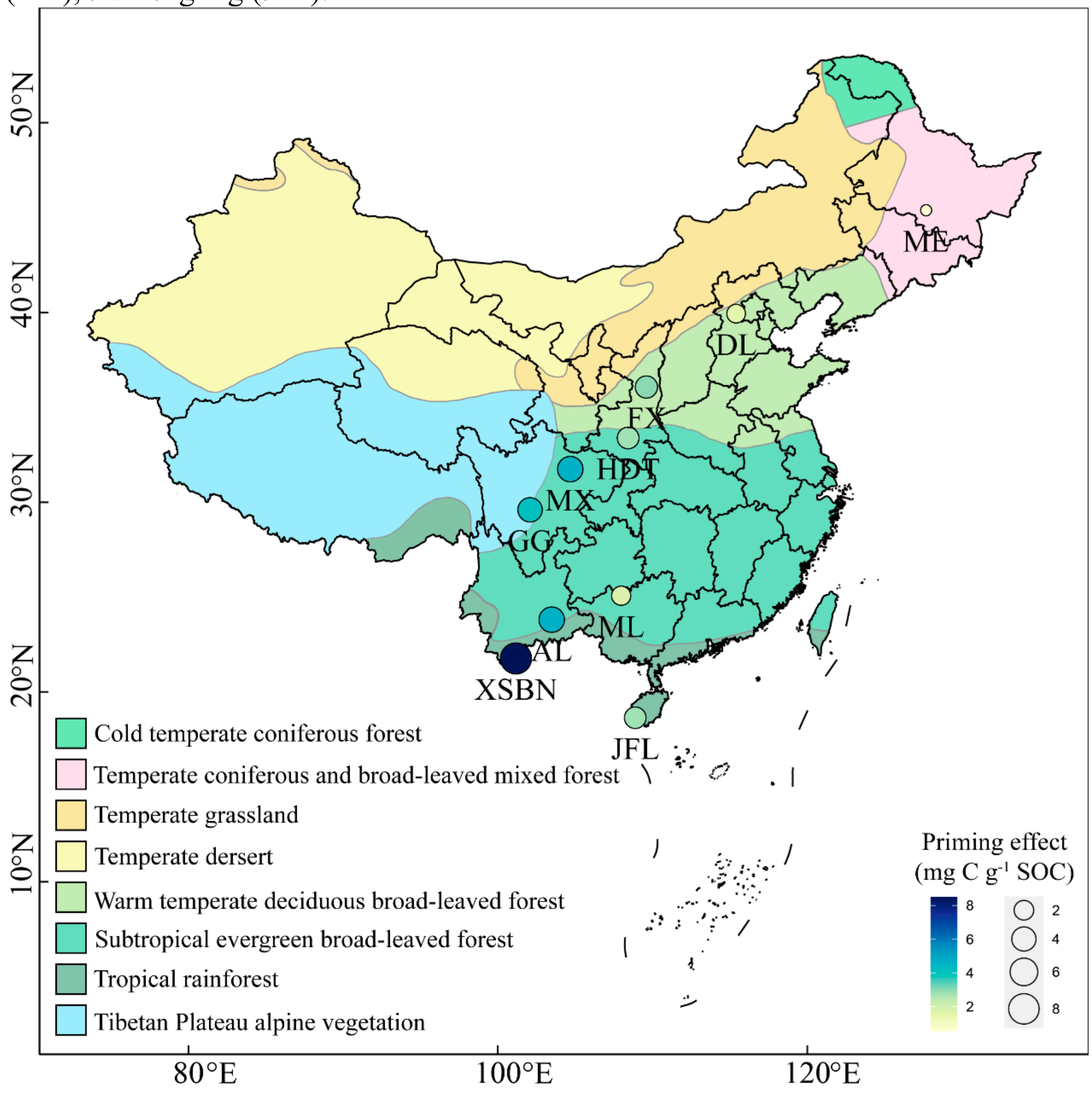

Supplement: Supplementary file 1 [file microorganisms-13-02799-s001.zip › microorganisms-3991538-supplementary.pdf]
